# Frailty and poor physical functioning as risk factors for driving cessation

**DOI:** 10.3389/fpubh.2024.1298539

**Published:** 2024-05-03

**Authors:** Thelma J. Mielenz, Haomiao Jia, Carolyn G. DiGuiseppi, David Strogatz, Howard F. Andrews, Lisa J. Molnar, David W. Eby, Linda L. Hill, Guohua Li

**Affiliations:** ^1^Department of Epidemiology, Mailman School of Public Health, Columbia University, New York, NY, United States; ^2^Center for Injury Science and Prevention, Columbia University Irving Medical Center, New York, NY, United States; ^3^Department of Biostatistics, Mailman School of Public Health, Columbia University, New York, NY, United States; ^4^Department of Epidemiology, Colorado School of Public Health, University of Colorado Anschutz Medical Campus, Aurora, CO, United States; ^5^Bassett Research Institute, Cooperstown, NY, United States; ^6^Department of Psychiatry, Vagelos College of Physicians and Surgeons, Columbia University, New York, NY, United States; ^7^University of Michigan Transportation Research Institute, Ann Arbor, MI, United States; ^8^School of Public Health, University of California San Diego, La Jolla, CA, United States; ^9^Department of Anesthesiology, Vagelos College of Physicians and Surgeons, Columbia University, New York, NY, United States

**Keywords:** older adults, driving cessation, frailty, physical performance, driving outcomes

## Abstract

**Introduction:**

Frailty and low physical performance are modifiable factors and, therefore, targets for interventions aimed at delaying driving cessation (DC). The objective was to determine the impact of frailty and physical performance on DC.

**Methods:**

Multisite prospective cohort of older drivers. The key inclusion criteria are as follows: active driver age 65–79 years, possessing a valid driver’s license, without significant cognitive impairment, and driving a 1996 car or a newer model car. Of the 2,990 enrolled participants, 2,986 (99.9%) had at least one frailty or Short Physical Performance Battery (SPPB) measure and were included in this study. In total, 42% of participants were aged 65–69 years, 86% were non-Hispanic white, 53% were female, 63% were married, and 41% had a high degree of education. The Fried Frailty Phenotype and the Expanded Short Physical Performance Battery (SPPB) from the National Health and Aging Trends Study were utilized. At each annual visit, DC was assessed by the participant notifying the study team or self-reporting after no driving activity for at least 30 days, verified via GPS. Cox proportional hazard models, including time-varying covariates, were used to examine the impact of the SPPB and frailty scores on time to DC. This assessment included examining interactions by sex.

**Results:**

Seventy-three participants (2.4%) stopped driving by the end of year 5. Among women with a fair SPPB score, the adjusted hazard ratio (HR) of DC was 0.26 (95% confidence interval (CI) 0.10–0.65) compared to those with a poor SPPB score. For those with a good SPPB score, the adjusted HR of DC had a *p*-value of <0.001. Among men with a fair SPPB score, the adjusted hazard ratio (HR) of DC was 0.45 (95% CI 0.25–0.81) compared to those with a poor SPPB score. For men with a good SPPB score, the adjusted HR of DC was 0.19 (95% CI 0.10–0.36). Sex was not an effect modifier between frailty and DC. For those who were categorized into pre-frail or frail, the adjusted ratio of HR to DC was 6.1 (95% CI 2.7–13.8) compared to those who were not frail.

**Conclusion and relevance:**

Frailty and poor physical functioning are major risk factors for driving cessation. Staying physically active may help older adults to extend their driving life expectancy and mobility.

## Introduction

Driving cessation (DC) decreases independence and leads to poorer physical, mental, and social health (1). Frailty and physical performance are both modifiable factors by behavioral factors such as progressive exercise interventions (2). The Short Physical Performance Battery (SPPB), a comprehensive lower extremity physical performance measure is a strong predictor of skilled nursing admission, morbidity, and mortality (3, 4). It has also been found to be a good measure of physical functioning and driving outcomes (3, 4). The SPPB has previously been assessed as a predictor of DC (4). Gill et al. found that low SPPB scores were associated with 120% of the hazard of DC [HR = 2.2, 95% CI 1.3, 3.7] compared to high scores (3, 5). However, this sample came from one health plan in one geographic region and was oversampled based on physical disability (5). The data from our study sample may yield results that are more generalizable than those from the study by Gill et al. (5).

While the SPPB provides a valuable measure of physical capabilities in terms of lower extremity physical function, frailty is a clinical syndrome that develops following a decline in function and resilience across numerous physiological systems. Therefore, frailty provides an alternative, more comprehensive measure of physical capabilities than the SPPB (6, 7). Frailty is associated with falls, disability, hospitalization, and injury mortality (6–8). Baseline frailty in this cohort has previously been found to be associated with the rate of DC at 1 year HR of 4.2 (95% CI: 1.9–9.1) for pre-frail and HR of 6.1 (95% CI: 1.4–27.3) for frail participants (8). Ishii et al. evaluated driving cessation as the exposure and physical frailty transition as the outcome in a cohort of 2,934 older Japanese adults (9). They reported that driving cessation was a risk factor for frailty, as indicated by a 4.6% versus 17.1% difference in the rates for frailty transition (9). Our study is the first to evaluate time-varying frailty as the exposure and driving cessation as the outcome longitudinally.

The aim of this study was to examine the impact of frailty and SPPB on the likelihood of DC. We hypothesized that the presence of frailty and poor SPPB would lead to a higher likelihood of DC.

## Methods

### Participants, design, and procedures

We utilized the AAA Longitudinal Research on Aging Drivers (LongROAD) cohort, and the methods were described in detail previously (10). Using a clinical convenience sample, 40,806 patients were approached, with 7.3% enrolling in the study. LongROAD was a multisite prospective cohort of 2,990 older drivers enrolling participants aged 65–79 years recruited between July 2015 and March 2017 from five sites: Ann Arbor, MI; Baltimore, MD; Cooperstown, NY; Denver, CO; and San Diego, *CA.* The inclusion criteria were as follows: (1) living at their current address 80% of the year, (2) possessing a valid driver’s license, (3) driving on average at least once per week, (4) having no significant cognitive impairment, (5) driving one vehicle ≥80% of the time, (6) driving a vehicle with model year 1996 or newer with an available OBDII port, (7) not involved in another driving study, (8) planning to stay in their current location for another 5 years, and (9) not living with a current LongROAD participant (10). The annual data collection was completed in the following date ranges: Baseline —6 July 2015 to 31 March 2017; Year 1—6 July 2016 to 20 June 2018; Year 2—21 June 2017 to 8 June 2019; Year 3—3 July 2018 to 30 April 2020; Year 4—25 June to 24 November 2021; and Year 5—31 August 2020 to 27 September 2022. The Baseline, Year 2, and Year 4 or 5 (due to COVID-19 half of the cohort in Year 4 and the other half of the cohort in Year 5) visits were conducted in person. The other visits were conducted by telephone. Participants classified by the Fried Frailty Phenotype (FFP) or SPPB at least once (*n* = 2,986, 99.9%) were included in this analysis.

### Measures

#### Covariates

It is well known that poor cognition and vision are important risk factors for driving cessation. Episodic and working memory were measured with immediate and delayed word recall tasks with higher scores indicative of better cognitive health (11). The Motor Free Visual Perception Test (MVPT-3) measured overall visual perception ability based on test items 22–34 (scores ranged from 0 to 13 with 13 indicating better visual perception) (12, 13).

#### Exposure

##### Short physical performance battery

Lower extremity physical function was measured using scores from the timed components of the National Health and Aging Trends Study (NHATS) Expanded SPPB: walking speed; repeated chair stands; three original standing balance tests (side-by-side, semi-tandem, and tandem); and an additional balance test (standing on one leg with eyes open) (14). Each participant received scores ranging from 0 to 4 for the three components. An SPPB summary score ranging from 0 to 12, with 12 representing the highest performance level, was created by summing the three scores. To account for a high level of performance in this cohort, cutoffs for each category were raised so that the SPPB scores are categorized as poor (0–7), fair (8–10), and good (11–12) (3).

#### Frailty

Frailty was measured using the FFP (6–8). Frailty status was evaluated by assessing five criteria on a pass/fail basis: shrinking (unintentional loss of ≥10 pounds in the past year or being underweight according to a body mass index (BMI) of ≤18.5 kg/m^2^), weakness (grip strength in the lowest 20% of the population, adjusted for sex and BMI), exhaustion (self-report of having poor endurance and energy), slowness (slowest 20% of the population based on time to walk 15 ft., adjusted for sex, and standing height), and low physical activity (not having recently walked for exercise or engaged in vigorous physical activity). If three or more of these criteria were met, the participant was classified as frail; if one or two criteria were met, the participant was classified as pre-frail; and if none was met, the participant was classified as not frail (6). In this study, the pre-frail and frail categories were collapsed into the “frail” category due to the models not converging with separate categories.

#### Primary outcomes

##### Driving cessation

Driving cessation was based on participants’ self-report, which was captured in one of the three ways. First, questions about driving status were assessed at each annual follow-up visit. Second, participants were instructed to notify the study team if they stopped driving. Third, participants’ driving activity was monitored using an in-vehicle GPS device. If there was no driving for at least 30 days, the study team reached out to participants to identify their current driving status.

### Statistical analysis

Cox proportional hazard (PH) models with time-varying covariates were used to examine the impact of annual SPPB and frailty with time to DC among those who were still driving. SPPB and frailty were examined in separate PH models. The model fits data from day 0 to the end of the follow-up for each person. Each model included the following covariates to control confounding of data: age, sex, marital status, education, delayed and immediate word recall, and MVPT-3. All these covariates (except for sex) were time-varying (The model estimates were adjusted for clustering within each site.). The models were tested for interaction by sex.

## Results

At baseline, 42% were 65–69 years old, 53% were female, 86% were non-Hispanic white, 41% had an advanced degree, and 63% were married (Table 1).

**Table 1:** Characteristics of baseline participants (*N* = 2,986).

Variables	*N*	%	Mean (SD)
Age (years)			
65–69	1,242	41.6%	
70–74	1,036	34.7%	
75–79	708	23.7%	
Sex			
Male	1,403	47.0%	
Female	1,583	53.0%	
Race/ethnicity			
White, non-hispanic	2,555	85.9%	
Black, non-hispanic	213	7.2%	
Other	206	6.9%	
Marital status			
Married	1873	63.3%	
Not married	1,085	36.7%	
Education			
High school or less	336	11.3%	
Associate’s degree	724	24.3%	
Bachelor’s degree	696	23.4%	
Advanced degree	1,221	41.0%	
SPPB			
0–7 (poor)	572	19.4%	
8–10 (fair)	1,350	45.8%	
11–12 (good)	1,025	34.8%	
Frailty			
Not frail	1,222	41.2%	
Pre-frail or frail	1742	58.8%	
Motor free visual perception test (1–13 higher better)			11.5 (1.7)
Immediate and delayed correct word recall			10.5 (3.0)

Seventy-three participants stopped driving by Year 5. One participant resumed driving after having stopped. The interaction between SPPB fair level and sex was not significant (*p* = 0.32), and the interaction between SPPB good level and sex was significant (*p* = 0.0001). Overall, the interaction between SPPB and sex was significant (*p* = 0.0271), thus we ran both combined and stratified models by sex. For the combined models, after controlling for the time-varying covariates and comparing fair SPPB to poor SPPB, the results were as follows: adjusted (age, sex, marital status, education. delayed and immediate word (NOTE: NOT WORK), and vision) and adjusting for clustering within each site, the hazard ratio (aHR) was 0.31 (95% confidence interval (CI) 0.18, 0.52). Comparing good SPPB to poor SPPB the aHR was 0.09 (95% CI 0.04, 0.20). Among men, after controlling for the time-varying covariates (age, marital status, education, delayed and immediate word recall, and vision) and sex and adjusting for clustering within each site, the comparison of fair SPPB to poor SPPB, yielded the following results: the adjusted hazard ratio (aHR) was 0.45 (95% confidence interval (CI) 0.25, 0.81). Comparing good SPPB to poor SPPB, the aHR was 0.19 (95% CI 0.10, 0.36). Among women, after controlling for the time-varying covariates (age, marital status, education, delayed and immediate work recall, and vision) and adjusting for clustering within each site, comparing fair SPPB to poor SPPB, the results were as follows: adjusted hazard ratio (aHR) was 0.26 (95% CI 0.10, 0.65). Comparing good SPPB to poor SPPB, the aHR was <0.001 (see Table 2). For FFP, sex was not a significant effect modifier in the relationship between frailty and driving cessation (*p* = 0.9810) (see Table 3).

**Table 2:** Unadjusted and adjusted association of short physical performance battery (SPPB) with time to driving cessation.

	Unadjusted hazard ratio (95% confidence interval)^1^	Adjusted hazard ratio (95% confidence interval)^2^
SPPB status		
Poor	Ref	Ref
Fair	0.29 (0.19–0.46)	0.31 (0.18–0.52)
Good	0.08 (0.05–0.14)	0.09 (0.04–0.20)

Male individuals	Unadjusted hazard ratio (95% confidence interval)^3^	Adjusted hazard ratio (95% confidence interval)^4^
SPPB status		
Poor	Ref	Ref
Fair	0.33 (0.14–0.80)	0.45 (0.25–0.81)
Good	0.08 (0.05–0.13)	0.19 (0.10–0.36)

Female individuals	Unadjusted hazard ratio (95% confidence interval)^5^	Adjusted hazard ratio (95% confidence interval)^4^
SPPB status		
Poor	Ref	Ref
Fair	0.28 (0.15–0.52)	0.26 (0.10–0.65)
Good	0.12 (0.04–0.35)	4.0 × 10^−8^ (1.5 × 10^−8^ – 1.1 × 10^−7^)

^1^Total *N* = 2,976. ^2^Adjusted for age, sex, marital status, education, delayed and immediate word recall, vision, and adjusting for clustering within each site. ^3^Total *N* = 1,358. ^4^Adjusted for age, marital status, education, delayed and immediate word recall, vision, and adjusting for clustering within each site. ^5^Total *N* = 1578.

**Table 3:** Unadjusted and adjusted association of frailty status with time to driving cessation.

	Unadjusted hazard ratio (95% confidence interval)^a^	Adjusted hazard ratio (95% confidence interval)^b^
Frailty Status		
Not Frail	Ref	Ref
Pre-frail and Frail	2.69 (1.73–4.16)	6.12 (2.72–13.78)

a Total *N* = 2978.

b Adjusted for age, gender, marital status, education, delayed and immediate word recall, and vision, and adjusting for clustering within each site.

After controlling for the time-varying covariates (age, marital status, education, delayed and immediate work recall, and vision) and sex and adjusting for clustering within each site, the comparison of pre-frail and frail individuals with those not frail resulted in a HR of 6.1 (95% CI 2.7, 13.8).

## Discussion

This study assessed the impact of repeated measures of frailty and SPPB and followed participants over 5 years for the likelihood of DC. Over the 5-year study period, we found a protective association of higher physical performance, especially in women, and a negative association of frailty with time to DC. Ishii et al. found that DC was an independent risk factor of physical frailty transition in older adults, but this is the first study to assess the impact of physical frailty transition on DC (9). Previous research utilized the baseline LongROAD data and evaluated SPPB with driving space and crashes, but did not find effect modification by sex (3). Gill et al. (5) assessed the risk factors associated with long-term disability in the community including driving a car and found that women had a HR of 1.86 (95% CI 1.41–2.46). Although sex differences in frailty are widely reported, we did not find sex differences while evaluating the time to driving cessation (15–17). It has been reported that women are more likely to transition into frailty and are more likely to change frailty status, and this should be evaluated in future studies (15, 18).

Frailty is not a static state and has been shown to be a dynamic state in a meta-analysis of 42,775 community-dwelling older adults, where 13.7% of them improved (15, 18). Targeted exercise interventions starting with balance and flexibility and then progressing to endurance and resistance training are shown to improve physical function in frail older adults when targeting the pre-frail (2, 15). More specifically, the American College of Sports Medicine guidelines suggest that resistance and balance training begin before endurance training (2, 19). Interventions incorporating exercise, nutrition, cognitive training, and behavioral therapy have shown sustained (6 months) improvements in frailty status (2, 20). Physical activity interventions prevented decline in on-road driving performance (21, 22). Age-related changes in both physical and cognitive function lead to driving cessation. These non-cognitive changes such as muscle strength are modifiable (23).

A limitation of this study is the low response rate to our clinical convenience sample, which may limit the generalizability of the findings to the entire US population of older drivers (3, 10). In the methods paper for LongROAD, Li et al. stated that the sample was overrepresented by non-Hispanic whites and individuals with higher education status (3, 10). In summary, improving both frailty and physical performance in older adults by keeping physically active, ensuring adequate nutrition, and undergoing cognitive training are interventions that older adults can focus on to potentially delay DC.

## Conclusion

Frailty and poor physical functioning impacted driving cessation. Future interventions should target frailty and poor physical functioning to prolong driving cessation.

## Data availability statement

The datasets presented in this article are not readily available because data access limited due to constraints from the consent form. Requests to access the datasets should be directed to tjm2141@cumc.columbia.edu.

## Ethics statement

The studies involving humans were approved by the institutional review boards of all participating institutions. The studies were conducted in accordance with the local legislation and institutional requirements. The participants provided their written informed consent to participate in this study.

## Author contributions

TM: Conceptualization, Data curation, Formal analysis, Funding acquisition, Investigation, Methodology, Project administration, Supervision, Writing – original draft, Writing – review & editing. HJ: Conceptualization, Formal analysis, Methodology, Supervision, Writing – original draft, Writing – review & editing. CD: Data curation, Funding acquisition, Investigation, Writing – review & editing. DS: Data curation, Funding acquisition, Investigation, Writing – review & editing. HA: Data curation, Funding acquisition, Investigation, Methodology, Writing – review & editing. LM: Data curation, Funding acquisition, Investigation, Methodology, Writing – review & editing. DE: Data curation, Funding acquisition, Investigation, Methodology, Writing – review & editing. LH: Data curation, Funding acquisition, Investigation, Writing – review & editing. GL: Data curation, Funding acquisition, Investigation, Methodology, Writing – review & editing.

## References

[1] Falkenstein M Karthaus M Brüne-Cohrs U. Age-related diseases and driving safety. Geriatrics (Basel). (2020) 5:80. doi: 10.3390/geriatrics5040080 33086572 PMC7709672

[2] Walston J Buta B Xue QL. Frailty screening and interventions: considerations for clinical practice. Clin Geriatr Med. (2018) 34:25–38. doi: 10.1016/j.cger.2017.09.004 29129215 PMC5726589

[3] Ng LS Guralnik JM Man C DiGuiseppi C Strogatz D Eby DW. Association of Physical Function with Driving Space and Crashes among Older Adults. Gerontologist. (2020) 60:69–79. doi: 10.1093/geront/gny178 30624694 PMC7317610

[4] Mielenz TJ Durbin LL Cisewski JA Guralnik JM Li G. Select physical performance measures and driving outcomes in older adults. Inj Epidemiol. (2017) 4:14. doi: 10.1186/s40621-017-0110-2 28459121 PMC5420549

[5] Gill TM Gahbauer EA Murphy TE Han L Allore HG. Risk factors and precipitants of long-term disability in community mobility: a cohort study of older persons. Ann Intern Med. (2012) 156:131–40. doi: 10.7326/0003-4819-156-2-201201170-00009 22250144 PMC3278794

[6] Fried LP Tangen CM Walston J Newman AB Hirsch C Gottdiener J. Cardiovascular health study collaborative research group. Frailty in older adults: evidence for a phenotype. J Gerontol A Biol Sci Med Sci. (2001) 56:M146–57. doi: 10.1093/gerona/56.3.m146 11253156

[7] Xue QL. The frailty syndrome: definition and natural history. Clin Geriatr Med. (2011) 27:1–15. doi: 10.1016/j.cger.2010.08.009 21093718 PMC3028599

[8] Crowe CL Kannoth S Andrews H Strogatz D Li G DiGuiseppi C. Associations of frailty status with low-mileage driving and driving cessation in a cohort of older drivers. Geriatrics (Basel). (2020) 5:19. doi: 10.3390/geriatrics5010019 32204350 PMC7151033

[9] Ishii H Doi T Tsutsumimoto K Nakakubo S Kurita S Shimada H. Driving cessation and physical frailty in community-dwelling older adults: a longitudinal study. Geriatr Gerontol Int. (2021):21. doi: 10.1111/ggi.14272 34532952

[10] Li G Eby DW Santos R Mielenz TJ Molnar LJ Strogatz D. Longitudinal research on aging drivers (LongROAD): study design and methods. Inj Epidemiol. (2017) 22:4. doi: 10.1186/s40621-017-0121-z PMC5537138 28736796

[11] Wallace RB Herzog AR. Overview of the health measures in the health and retirement study. J Hum Res. (1995) 30:S84–S107. doi: 10.2307/146279

[12] Eby DW Molnar LJ Kostyniuk LP Zakrajsek JS Ryan L Zanier N. The association between visual abilities and objectively-measured driving space, exposure, and avoidance among older drivers: a preliminary analysis. J Aust College Road Safety. (2018) 29:39–45. doi: 10.3316/informit.146311795135973

[13] Coarusso RP Hammill DD. Motor-free Visual Perception Test-Third Edition. Novato, C A: Academic Therapy Publications (2003).

[14] Kasper J. D. Freedman V. A. Niefeld M. R. (2012). Construction of performance-based summary measures of physical capacity in the National Health and aging trends study. NHATS technical paper #4. Available at: https://www.nhats.org/scripts/TechnicalPerfSumMeasure.htm

[15] Kojima G Taniguchi Y Iliffe S Jivraj S Walters K. Transitions between frailty states among community-dwelling older people: a systematic review and meta-analysis. Ageing Res Rev. (2019) 50:81–8. doi: 10.1016/j.arr.2019.01.010 30659942

[16] Hubbard RE. Sex differences in frailty. Interdiscip Top Gerontol Geriatr. (2015) 41:41–53. doi: 10.1159/000381161 26301978

[17] Collard RM Boter H Schoevers RA Oude Voshaar RC. Prevalence of frailty in community-dwelling older persons: a systematic review. J Am Geriatr Soc. (2012) 60:1487–92. doi: 10.1111/j.1532-5415.2012.04054.x 22881367

[18] Trevisan C Veronese N Maggi S Baggio G Toffanello ED Zambon S. Factors influencing transitions between frailty states in elderly adults: the Progetto Veneto Anziani longitudinal study. J Am Geriatr Soc. (2017) 65:179–84. doi: 10.1111/jgs.14515 27861714

[19] American College of Sports Medicine Chodzko-Zajko WJ Proctor DN Fiatarone Singh MA Minson CT Nigg CR. American College of Sports Medicine position stand. Exercise and physical activity for older adults. Med Sci Sports Exerc. (2009) 41:1510–30. doi: 10.1249/MSS.0b013e3181a0c95c 19516148

[20] Ng TP Feng L Nyunt MS Feng L Niti M Tan BY. Nutritional, physical, cognitive, and combination interventions and frailty reversal among older adults: a randomized controlled trial. Am J Med. (2015) 128:1225–1236.e1. doi: 10.1016/j.amjmed.2015.06.017 26159634

[21] Marottoli RA Allore H Araujo KL Iannone LP Acampora D Gottschalk M. A randomized trial of a physical conditioning program to enhance the driving performance of older persons. J Gen Intern Med. (2007) 22:590–7. doi: 10.1007/s11606-007-0134-3 17443366 PMC1852916

[22] Abe T Fujii K Seol J Fujii Y Joho K Sato A. Driving frequency associated with deficits in lower extremity function, dynamic vision, and physical activity in Japanese older adults. J Trans Health. (2018) 9:282–7. doi: 10.1016/j.jth.2018.01.010

[23] Maliheh A Nasibeh Z Yadollah AM Hossein KM Ahmad D. Non-cognitive factors associated with driving cessation among older adults: an integrative review. Geriatr Nurs. (2023) 49:50–6. doi: 10.1016/j.gerinurse.2022.10.022 36435172

